# Compliance with Home-Based Prehabilitation and Length of Stay After Total Hip Arthroplasty: A Prospective Cohort Study

**DOI:** 10.3390/jcm15103898

**Published:** 2026-05-19

**Authors:** Paweł Hereć, Jakub Mazur, Robert Fiut, Weronika Wasyluk, Alicja Wójcik-Załuska, Jacek Gągała

**Affiliations:** 1 Department of Clinical Physiotherapy, Medical University of Lublin, ul. Jaczewskiego 8, 20-954 Lublin, Poland; pawel.herec@umlub.edu.pl (P.H.); jakubmazurfizjo@gmail.com (J.M.); alicja.wojcik-zaluska@umlub.edu.pl (A.W.-Z.); 2 Department of Internal Medicine and Internal Nursing, Medical University of Lublin, ul. Chodźki 7, 20-093 Lublin, Poland; weronika.wasyluk@umlub.edu.pl; 3 Department of Orthopaedics and Traumatology, Medical University of Lublin, ul. Jaczewskiego 8, 20-954 Lublin, Poland; jacek.gagala@umlub.edu.pl

**Keywords:** arthroplasty, patient compliance, exercise therapy, length of stay, recovery of function

## Abstract

**Background/Objectives:** Patients awaiting total hip arthroplasty (THA) may have a preoperative period for home-based exercise. However, the benefit of prehabilitation may depend on programme completion. This study assessed the association between compliance with home-based prehabilitation and postoperative course after THA, particularly hospital stay and self-assessed health status at discharge, and explored associations between compliance and changes in clinical and functional outcomes. **Methods:** In this prospective single-centre observational cohort pilot study, 40 adults scheduled for elective THA were included in a planned 60-day home-based prehabilitation programme as standard preoperative care. Assessments were performed before prehabilitation, preoperatively, and at discharge. Compliance was recorded using a daily checklist and expressed as a compliance index. Associations were analysed using non-parametric tests and Spearman correlation. **Results:** Median compliance index was 32.41%. Higher compliance was observed in participants reporting improvement or marked improvement at discharge than in those reporting slight improvement or no improvement (*p* = 0.0076). Compliance was inversely correlated with postoperative length of stay, median 6 days (rho = −0.593, *p* < 0.001). Compliance was lower in participants who reported pain during exercise (*p* = 0.0127). No significant associations were found between compliance and postoperative symptoms or changes in hip muscle strength, mechanical muscle properties, pain intensity, or functional test performance between baseline and preoperative assessments. **Conclusions:** Greater compliance with home-based prehabilitation was associated with shorter postoperative hospitalization and more favorable self-assessed health status at discharge. These findings support strategies to improve programme completion and minimize exercise-related pain.

## 1. Introduction

Total hip arthroplasty (THA) is among the most commonly performed orthopaedic procedures. Recent registry-based evidence confirms the increasing global burden of joint arthroplasty. In a systematic review including data from 15 national arthroplasty registries and 209 annual reports, THA incidence increased by 130% to 210% between 2010 and 2023, while projections to 2050 indicate a further increase in THA volumes by 121% to more than 200% across analysed countries. This growing demand highlights the need for strategies that may optimise perioperative care and healthcare resource use, including prehabilitation [1]. It is indicated in patients with hip osteoarthritis who report persistent chronic pain and substantial functional limitation despite unsuccessful conservative treatment [2]. The perioperative period imposes a considerable metabolic burden, therefore the preoperative waiting time may be used to enhance muscle strength and functional performance, which are frequently considered predictors of postoperative outcomes in orthopaedic populations [3]. Prehabilitation denotes structured preoperative preparation using targeted interventions, including nutritional management, respiratory exercises, exercise training, educational and psychological support, smoking cessation and alcohol reduction strategies, and pharmacological management [2,4].

Implementing prehabilitation may improve preoperative functional status and potentially increase tolerance to the physiological burden of surgery and the perioperative period [2]. Interventional studies indicate that targeted preoperative programmes can enhance functional outcomes and muscle strength. Hermann et al. reported significant improvements in functional performance and muscle strength following preoperative progressive explosive resistance training [5]. Fernandes et al. showed that a supervised neuromuscular exercise programme before THA improved quality of life before and after surgery without increasing total intervention costs [6]. Programmes lasting longer than eight weeks have been associated with significant improvements across assessed physical functioning parameters, suggesting a dose-response relationship [2]. In a Cochrane review, Khan et al. found that multidisciplinary prehabilitation after THA was associated with fewer postoperative symptoms and a shorter length of hospital stay compared with standard hospital care [7]. However, the relationship between actual programme compliance and postoperative outcomes remains insufficiently explored, particularly in home-based prehabilitation settings, where supervision is limited and adherence may vary substantially.

The primary aim of the study was to assess the association between compliance with a home-based prehabilitation programme and the postoperative course following total hip arthroplasty, with particular emphasis on length of hospital stay and self-assessed health status at discharge. The secondary aims were to explore associations between compliance and postoperative symptoms, as well as changes in clinical and functional outcomes between baseline and the preoperative assessment.

## 2. Materials and Methods

### 2.1. Ethical Statement

All study procedures adhered to the principles of the Declaration of Helsinki and were performed in line with the requirements of the Bioethics Committee of the Medical University of Lublin. Ethical approval was granted on 22 May 2025 (approval code: KB-0024/81/05/2025). Written informed consent was obtained from each participant prior to study inclusion.

### 2.2. Study Design

Patients awaiting elective THA were recruited between June 2025 and December 2025 at the Orthopaedic Outpatient Clinic of University Clinical Hospital No. 4 in Lublin. The study was initiated in June 2025, baseline assessments started in July 2025, and elective THA procedures in the analysed cohort were performed between September and December 2025. During this surgical period, 146 patients were scheduled for elective THA at the study centre. Consecutive patients scheduled for elective THA were considered for inclusion only if their planned surgery date was known at least two months in advance, allowing completion of the planned 60-day home-based prehabilitation programme, and if they met the predefined eligibility criteria. Patients who could not complete the full 60-day programme before surgery were not included in the observational analysis, although they could receive shorter prehabilitation as part of routine standard care. Eligibility was verified using medical history, medical record review, and an initial functional examination. This prospective single-centre observational cohort pilot study included patients undergoing a routine home-based prehabilitation programme offered as part of standard preoperative care at the study centre. Only individuals who provided written informed consent for participation in the observational study were enrolled. All enrolled participants were analysed regardless of the level of programme completion. As this was an exploratory pilot study designed to generate preliminary estimates and assess feasibility rather than to test predefined hypotheses, no formal sample size calculation was performed. A sample of 40 participants was considered sufficient to provide preliminary estimates of the associations between programme compliance and postoperative outcomes and to assess the feasibility of the study procedures.

Assessments were performed at three time points (M1, baseline before prehabilitation, M2, immediately before THA after completion of the preoperative prehabilitation period, M3, postoperative assessment at discharge). At M1 and M2, muscle strength, mechanical muscle properties, functional performance, pain intensity, and patient-reported hip-related outcomes were assessed using predefined instruments (handgrip dynamometer, ActivForce 2, MyotonPRO., Timed Up and Go [TUG], 10-m Walk Test [10MWT], Visual Analogue Scale [VAS], Hip disability and Osteoarthritis Outcome Score [HOOS]) [8]. At M3, postoperative outcomes were collected via questionnaire, and clinical data were extracted from medical records. Detailed measurement procedures, including device settings, anatomical landmarks, test positions, and scoring procedures for MyotonPRO assessment [9]. (Table S1), isometric hip muscle strength assessment with ActivForce 2 (Table S2) [10,11], questionnaire items and coding (Table S3), and the home-based exercise programme (Table S4), are provided in the Supplementary Materials.

To minimise potential sources of bias, including observer and decision-related bias, all participants received standardised instructions regarding the routine prehabilitation programme, outcome assessments were performed according to predefined procedures, and data from the compliance checklists were reviewed for completeness and internal consistency at the preoperative visit. In addition, the study procedures were conducted independently of routine clinical decision-making. Orthopaedic clinicians responsible for postoperative management and discharge decisions were not members of the research team collecting or analysing compliance data. Compliance data were not analysed during the hospitalisation period, and summary measures were calculated only after completion of the pilot study. Therefore, discharge decisions were not influenced by the compliance assessment. The follow-up period ended at hospital discharge, which constituted the final assessment time point in the study. This study was reported in accordance with the STROBE statement for observational studies.

### 2.3. Eligibility Criteria for the Study

Inclusion criteria were hip osteoarthritis as the indication for scheduled elective THA at the study centre, a planned surgery date allowing completion of the full home-based prehabilitation period according to the protocol, age 18 years or older, and provision of written informed consent.

Exclusion criteria were pregnancy, haemodynamic instability, severe anaemia (haemoglobin < 6 g/dL), advanced musculoskeletal disorders or advanced neurological disorders, as well as other conditions in which moderate-intensity exercise was contraindicated, in particular uncontrolled diabetes or uncontrolled arterial hypertension. In addition, patients were excluded if they had contraindications to safe moderate-intensity exercise due to an unstable cardiac condition or decompensated cardiovascular disease, including, for example, symptomatic heart failure, unstable coronary artery disease, significant arrhythmias, or a recent cardiovascular event. Patients with exacerbations of chronic diseases that could increase the risk of exercise-related complications were also excluded. Furthermore, patients were excluded if they had significant orthopaedic limitations not directly related to hip disease that prevented safe performance of the prescribed exercises or functional tests, and individuals in whom the planned perioperative management or clinical status did not allow the assessments to be conducted according to the study protocol.

### 2.4. Questionnaire Instruments and Self-Reported as Well as Clinical Variables

Questionnaire data were collected using an author-developed questionnaire, self-completed according to standardised instructions. The questionnaire was administered at three time points. At M1, sex, age, cigarette smoking, marital status, place of residence, education level, duration of hip pain, operated side, history of joint arthroplasty, and self-rated preoperative motivation were recorded. In this study, sex referred to biological sex and was recorded as female or male. Gender-related variables were not collected. At M2, self-rated preparedness for surgery, patient-reported compliance with the exercise programme, and pain during exercises were recorded. At M3, the self-assessment of current health status relative to the preoperative status was recorded. Selected postoperative clinical data were obtained from medical records, including length of hospital stay, postoperative day of first mobilisation, and postoperative symptoms documented during hospitalisation (pain, swelling, fever). All questionnaire items used in the analysis, together with response options and analytical coding, are summarised in the Supplementary Materials (Table S3).

### 2.5. Physiotherapy Protocol

The intervention comprised a planned 60-day home-based preoperative exercise programme to be performed daily using written instructions and visual materials. Before initiation, a physiotherapist provided standardised instruction on safe technique and exercising within a clinically tolerated range. Sessions lasted approximately 20 min. Participants were advised to exercise at moderate intensity, keeping pain at or below 3 points on the 10-point VAS. If pain reached 4 points or higher during an exercise, participants were instructed to stop that exercise and modify the load.

Each session included strengthening and stretching exercises. Strengthening exercises were prescribed as 10 repetitions per exercise and included isometric quadriceps setting, hip flexion of the lower limb, hip bridge, straight leg raise at the hip joint, hip external rotation in side-lying, gluteal strengthening in standing, and half squats using a chair. Stretching exercises included hip rotations in supine, hip adductor stretch, and hip flexor stretching in a standing forward and backward lunge position using a chair. For unilateral exercises, sets were performed for both lower limbs. Rest breaks of 30 to 60 s were recommended between exercises, with longer breaks permitted when needed, and sessions could be split into two shorter blocks per day. Participants were advised to stop exercising and contact the research team if pain exceeding 4 points on the VAS persisted despite modifications, or if new or worsening symptoms occurred, including dizziness, dyspnoea, chest pain, palpitations, neurological symptoms, fever, or marked swelling. Instructional materials, including exercise descriptions and illustrations, are provided in the Supplementary Materials (Table S4). The programme was developed based on published prehabilitation protocols [12,13].

### 2.6. Handgrip Dynamometry

Handgrip strength (HGS) was assessed using a hydraulic dynamometer (Saehan SH5001, Saehan Medical Co., Ltd., Changwon, Republic of Korea) according to a standard protocol. Participants were seated with the elbow flexed to 90 degrees, the forearm in a neutral position, and the tested upper limb held away from the trunk. After instruction, maximal grip was performed on the command “start” and terminated on “stop”. Three trials were conducted for each hand with short rest breaks, and the highest value was used for analysis, regardless of side [14,15]. The dynamometer was disinfected before each use. Percentile categories were assigned using baseline (M1) HGS values relative to sex- and age-specific reference norms and classified as ≤10th, 10th–25th, 25th–50th, 50th–75th, 75th–90th, and ≥90th percentile [14].

### 2.7. Visual Analogue Scale

Pain intensity was assessed using the VAS, a 10 cm horizontal line anchored by “no pain” and “worst imaginable pain”. Participants marked their pain intensity on the line, and the score was calculated as the distance (cm) from the “no pain” anchor to the mark. The VAS template is provided in the Supplementary Materials (Figure S1) [16].

### 2.8. Compliance Assessment

Compliance with the prehabilitation programme was monitored using a daily participant-completed checklist. The daily score reflected the participant-reported proportion of the prescribed exercise programme completed on a given day, rather than the number of individual exercises analysed separately. The compliance index was calculated as the arithmetic mean of all daily completion scores across the planned 60-day period, with each day assigned equal weight, and expressed as a percentage, with missing entries coded as 0%. The checklist template is provided in the Supplementary Materials (Figure S2).

### 2.9. Statistical Analysis

Statistical analyses were performed using Statistica (v. 13 PL; TIBCO Software Inc., Palo Alto, CA, USA) and MedCalc (v. 23.4.8; MedCalc Software Ltd., Ostend, Belgium). Normality was assessed with the Shapiro-Wilk test. As most variables were non-normally distributed, non-parametric methods were applied. Categorical variables were reported as *n* (%), and continuous variables as median (Q1; Q3). Group comparisons were performed using the Mann-Whitney U test (two independent groups) or the Kruskal-Wallis test (more than two independent groups). Associations between continuous variables were assessed using Spearman’s rank correlation. Box-and-whisker plots and scatter plots were used for data visualisation. No missing data were identified for the variables included in the main analyses, and all enrolled participants completed the assessments analysed at M2 and M3. A two-sided *p* value < 0.05 was considered statistically significant.

## 3. Results

### 3.1. Participant Flow

Of the 146 patients scheduled for elective THA during the surgical period, 95 did not meet the timing requirement for the planned 60-day observational protocol and were therefore not approached for participation in the observational analysis. The remaining 51 met the timing requirement for the planned 60-day observational protocol and were approached for participation. Eleven declined participation at the recruitment stage when contacted by telephone because of logistical difficulties related to travel distance to the study centre. The remaining 40 patients were assessed for eligibility, none were excluded, and all enrolled participants completed the assessments at M1, M2, and M3. No missing data were identified for the variables included in the main analyses.

### 3.2. Patient Characteristics

A total of 40 patients awaiting THA were included, and all procedures were performed using a posterolateral approach. The sample comprised 25 (62.5%) men and 15 (37.5%) women. Median age was 63.5 years, median body weight was 83.5 kg, median body mass index (BMI) was 29.36 kg/m^2^, and median waist circumference was 100 cm. Current cigarette smoking was reported by 8 patients (20%). Surgery more often involved the left hip than the right hip (57.5% vs. 42.5%). Previous hip arthroplasty was reported by 11 (27.5%) patients, while 29 (72.5%) patients reported none.

Self-rated motivation and preparedness were most frequently rated as 5 points on a 1 to 5 scale (47.5% and 50%, respectively). At M2, 23 patients (57.5%) declared completion of the exercise programme, and 25 (62.5%) reported pain during exercises. The median compliance index was 32.41% (Q1; Q3: 0.00; 72.90) (Table 1).

HGS at the first assessment was 21.0 kg in women and 52.0 kg in men. In women, the distribution across HGS percentile categories was as follows: 6.7% were at or below the 10th percentile, 26.7% between the 10th and 25th percentile, 20% between the 25th and 50th percentile, 26.7% between the 50th and 75th percentile, 13.3% between the 75th and 90th percentile, and 6.7% at or above the 90th percentile. In men, 12% were at or below the 10th percentile, 4% between the 10th and 25th percentile, 12% between the 25th and 50th percentile, 28% between the 50th and 75th percentile, 24% between the 75th and 90th percentile, and 20% at or above the 90th percentile.

### 3.3. Compliance and Postoperative Course

**Figure 1:**
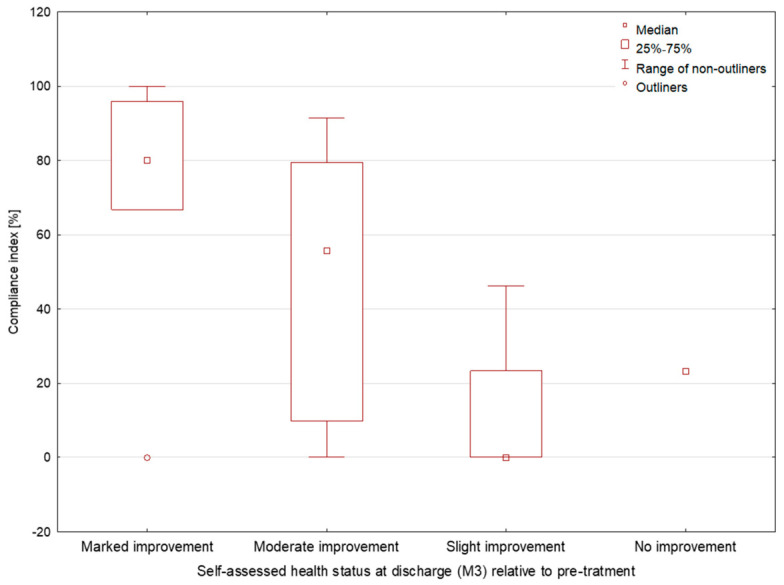
Compliance index according to self-assessed health status at discharge relative to the pre-treatment status.

**Figure 2:**
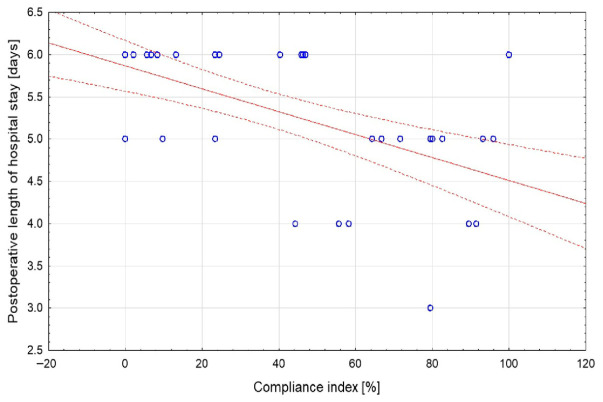
Correlation between compliance index and postoperative length of hospital stay. Dots represent individual participants, and the dashed line represents the fitted regression line.

The compliance index was significantly higher in participants who reported moderate improvement or marked improvement in current health status compared with those who reported slight improvement or no improvement (55.7% and 80% vs. 0% and 23.3%, *p* = 0.0076, Figure 1). The median postoperative length of hospital stay was 6 days (5; 6). A statistically significant negative correlation was found between the compliance index and postoperative length of hospital stay (rho = −0.593, *p* < 0.001, Figure 2), indicating that higher compliance was associated with a shorter postoperative hospital stay. Detailed data are presented in Table 2.

### 3.4. Compliance and Changes in Clinical and Functional Outcomes

No statistically significant associations were found between the compliance index and changes in clinical or functional outcomes from M1 to M2. Spearman’s rank correlations between compliance index and changes in HOOS domains, VAS pain intensity, MyotonPRO parameters, isometric hip muscle strength, and functional test performance (TUG, 10MWT) were small and non-significant. Detailed correlation coefficients and *p* values are provided in the Supplementary Materials (Table S5).

### 3.5. Compliance and Demographic and Clinical Characteristics

**Figure 3:**
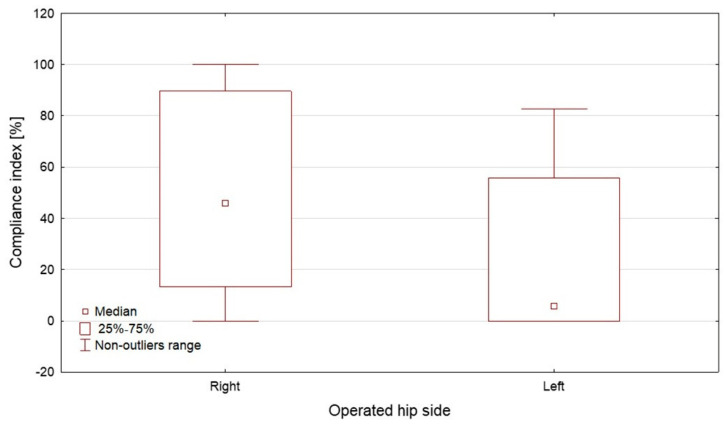
Compliance index according to operated hip side.

**Figure 4:**
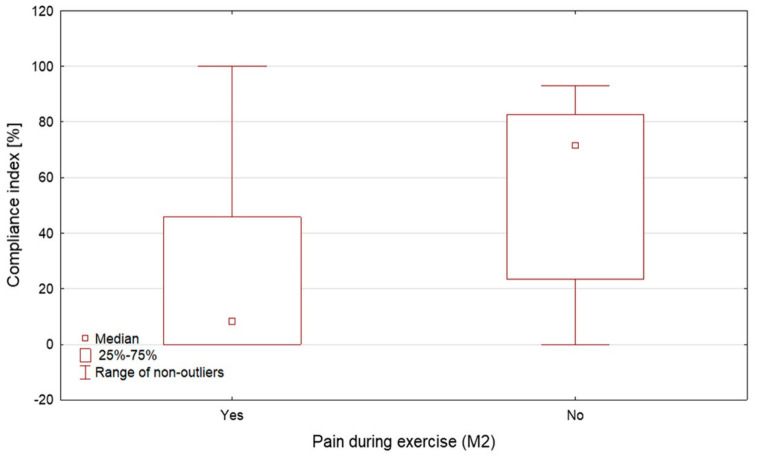
Compliance index according to pain during exercise at M2.

Compliance index was significantly higher in participants scheduled for right hip surgery than in those scheduled for left hip surgery (45.8% vs. 5.7%, *p* = 0.0177, Figure 3) and in participants who did not report pain during exercises compared with those who reported pain (71.7% vs. 8.3%, *p* = 0.0127, Figure 4). A non-significant trend toward higher compliance was observed in women compared with men (46.3% vs. 9.7%, *p* = 0.0578). Detailed associations with demographic and clinical variables are provided in the Supplementary Materials (Table S6).

### 3.6. Compliance and Self-Rated Programme Completion

Participants who self-reported completing the programme had a significantly higher compliance index than those who reported not completing the programme (64.3% vs. 0.0%, *p* < 0.001). The median compliance index was 64.3% (44.7; 82.0) in participants who declared programme completion and 0% (0.0; 7.1) in those who declared non-completion.

## 4. Discussion

In this prospective single-centre observational cohort pilot study, with assessments at M1 and M2 and outcome analyses at M3, we examined associations between the degree of compliance with a home-based exercise prehabilitation programme and selected functional and clinical parameters in patients scheduled for elective THA. Substantial variability in exercise programme completion was observed in the study group. Pain reported during exercises emerged as an important barrier to programme completion and was associated with lower compliance. Compliance index was associated with a more favourable self-assessment of health status after surgery and a shorter postoperative length of hospital stay. These findings suggest that potential benefits of prehabilitation may depend to a considerable extent on actual exposure to the intervention. Therefore, strategies aimed at improving compliance, together with appropriate pain management, should be considered key components in the design and evaluation of preoperative programmes. The median compliance index was 32.41% with a planned prehabilitation duration of 60 days. Compared with the available literature, this indicates a relatively low level of programme completion. In published studies, prehabilitation has most often been delivered in an unsupervised format, and compliance has shown considerable variability, ranging from 16% to 97%. Moreover, some publications do not report compliance, which limits interpretation of intervention effectiveness. Feasibility reports also indicate implementation barriers, including time constraints, which result in a substantial proportion of patients being ineligible for participation, while the proportion consenting to recruitment remains low. Consequently, incomplete data on participation and programme completion may reduce the generalisability of findings from prehabilitation research [17]. In a systematic review with meta-analysis of randomised controlled trials on prehabilitation, 11 studies reported compliance. Within this subgroup, high programme completion was observed, with a mean compliance of 90.5% [18].

In the present study, the compliance index was higher in participants who did not report pain during exercises than in those who reported pain. This is consistent with reports indicating that pain, often accompanied by fatigue and stiffness, represents an important barrier to physical activity in individuals with osteoarthritis, limiting the ability to perform prescribed exercises and reinforcing a vicious cycle in which more severe symptoms reduce activity, while reduced activity may secondarily exacerbate symptoms and further avoidance of exercise. With disease progression and concomitant excess body mass, symptoms may additionally reduce exercise tolerance and perceived agency. Subjectively perceived lower fitness, older age, and low physical performance may strengthen the belief that the programme requirements cannot be met, which in practice may promote selective performance of exercises or discontinuation [19].

In the present study, self-reported programme completion was broadly consistent with the obtained quantitative compliance indices. Participants who described the programme as completed achieved a higher compliance index than those who reported non-completion. However, interpretation of this association should consider limitations of self-report methods. Validation studies have shown that exercise diaries may overestimate programme performance compared with objective measurement, while agreement between methods is moderate and characterised by substantial interindividual variability. In addition, self-report may have limited reliability. For these reasons, interpretation of prehabilitation programme completion should be based primarily on quantifiable numerical indicators rather than solely on participant declarations, which may improve the validity of conclusions regarding the relationship between the “dose” of exercise and clinical outcomes [20].

The literature has reported a preoperative improvement in TUG of approximately 2.9 s, with a 95% confidence interval that included both no effect and a possible improvement, ranging from −0.9 to 6.6 s. In our study, no statistically significant differences in TUG were found. This may be consistent with the observation above, as a wide confidence interval indicates uncertainty of the effect estimate, likely related to limited statistical power and heterogeneity of participants and interventions. From a clinical perspective, it is therefore appropriate to interpret TUG results not only in terms of *p* values, but also in terms of effect size, confidence intervals, and whether the observed change may be functionally meaningful in a given population [21]. Consistent with our observations, Rooks et al. also did not demonstrate a significant difference in TUG [22].

A meta-analysis of randomised trials demonstrated a reduction in postoperative length of hospital stay after total knee arthroplasty in patients receiving prehabilitation, with low heterogeneity across analyses [18]. In our THA study, a shorter hospital stay was also observed, which is consistent with the direction of effect reported in the meta-analysis and may suggest that better functional preparation before surgery supports a more efficient early postoperative course. Moreover, Rooks et al. showed that a preoperative intervention was associated with a higher likelihood of discharge home rather than discharge to an inpatient rehabilitation facility, suggesting that potential benefits of prehabilitation may include not only shorter hospitalisation, but also a more favourable early postoperative rehabilitation pathway [22].

In our analysis, higher compliance index was observed in participants scheduled for right hip THA compared with those scheduled for left hip THA (45.8% vs. 5.7%, *p* = 0.0177), although left hip surgery was more frequently planned in the studied sample. This finding should be interpreted with caution, because in a review of the available literature on prehabilitation before hip arthroplasty we did not identify studies in which the side of the planned procedure was routinely evaluated as a predictor of programme completion. Comparisons across studies are additionally limited by substantial heterogeneity of interventions and methods used to measure exercise completion. Consequently, it cannot be determined whether the observed association is causal or reflects sample-specific characteristics, confounding, or random variation within exploratory analyses. Therefore, the observation regarding the planned surgery side should be considered hypothesis-generating and requires verification in larger studies, preferably using multivariable models that control for clinical and behavioural factors such as pain during exercises, baseline functional status, motivation, and self-efficacy.

The limitations of this study primarily result from its pilot design, small sample size, and the selection process required by the planned 60-day prehabilitation protocol. This exploratory study was designed to generate preliminary estimates and assess feasibility rather than test predefined hypotheses. Accordingly, the sample size was arbitrarily determined and may limit generalisability. Importantly, only patients whose planned surgery date allowed completion of the full 60-day programme could be included in the observational analysis. Therefore, patients with shorter waiting times before THA were not represented in the analysed cohort, although they could receive shorter prehabilitation as part of routine standard care. This timing-related criterion may have introduced selection bias and limits the applicability of the findings to the broader population of patients awaiting elective THA. In addition, 11 of 51 patients who met the timing requirement declined participation, mainly because of logistical difficulties related to travel distance to the study centre, which may have further selected for participants who were more able or willing to engage with the programme. In addition, compliance was assessed using a self-reported checklist, which may be subject to recall bias and social desirability bias, potentially leading to overestimation of actual programme completion. Interpretation of associations between compliance and clinical or functional outcomes also requires consideration of potential confounders. Furthermore, the absence of a control group limits the ability to distinguish compliance-related associations from natural history, usual postoperative recovery, or measurement variability. Compliance may reflect not only actual exposure to the intervention, but also participant characteristics, including motivation to engage in physical activity, self-efficacy, and readiness to change health behaviours. An additional limitation is the impact of pain during exercises, which may reduce the “dose” of training independently of participant intent, while also serving as a marker of more severe symptoms and worse baseline status. Another limitation is that the home-based prehabilitation programme did not include formal progression of external load, number of repetitions, or exercise difficulty, which may have limited the potential to induce measurable improvements in muscle strength and functional outcomes during the preoperative period. Furthermore, baseline function may modify both the ability to perform exercises and the potential to improve over time, which increases the risk of regression to the mean. Future studies should implement programme monitoring based on standardised recording of attendance and session duration within a supervised system, performed by staff delivering the intervention. Such an approach may reduce reporting bias and improve the credibility of evaluating associations between the “dose” of exercise and clinical outcomes.

## 5. Conclusions

Compliance with the home-based prehabilitation programme showed considerable variability, and pain reported during exercises constituted an important barrier limiting intervention implementation. Higher programme compliance was associated with a more favourable self-assessment of postoperative health status and a shorter postoperative length of hospital stay, which indicates the importance of actual exposure to the intervention for the observed outcomes. Self-reported programme completion was broadly consistent with measurable indicators of programme performance. Given the potential influence of baseline factors on both programme compliance and postoperative outcomes, analyses should account for key covariates, including baseline function and pain intensity. These observations support the design of future studies with larger samples and a more controlled design, preferably multicentre, with an emphasis on improving programme compliance through individualisation of training load, appropriate pain management, and standardised monitoring of attendance and session duration.

## Figures and Tables

**Table 1:** Characteristics of the study group. Values are presented as median (Q1; Q3) or *n* (%).

Variable	Study Group (*n* = 40)
Age, years	63.5 (57.34; 71.66)
Smoking, yes	8 (20%)
Body weight, kg	83.5 (80.00; 91.32)
BMI, kg/m^2^	29.36 (27.34; 30.85)
Waist circumference, cm	100 (90.68; 105.00)
Operated hip, left	23 (57.5%)
History of hip arthroplasty, yes	11 (27.5%)
Programme completion (self-report), yes	23 (57.5%)
Pain during exercise, yes	25 (62.5%)
Compliance index, %	32.41 (0.00; 72.90)

**Table 2:** Associations between the compliance index and categorical postoperative outcomes in the study group. Postoperative outcomes and the compliance index are presented as n and median (Q1; Q3), respectively.

Variable	Category	Postoperative Outcome, *n* (%)	Compliance Index [%]	*p*
Current health status compared with the pre-treatment status	marked improvement	7 (17.5%)	80.0 (66.8; 95.8)	0.0076 *
	moderate improvement	17 (42.5%)	55.7 (9.7; 79.5)	
	slight improvement	15 (37.5%)	0.0 (0.0; 23.4)	
	no improvement	1 (2.5%)	23.3 (23.3; 23.3)	
	deterioration	0	N/A	
Postoperative symptoms	no symptoms	28 (70%)	24.0 (0.0; 65.5)	0.2880
	pain	11 (27.5%)	40.3 (10.6; 77.5)	
	swelling	1 (2.5%)	0.0 (0.0; 0.0)	
	fever	0 (0%)	N/A	

* Statistically significant (*p* < 0.05).

## Data Availability

The data presented in this study are available on reasonable request from the corresponding author. Restrictions apply because the data contain potentially identifiable clinical information.

## Supplementary Materials

The following supporting information can be downloaded at: https://www.mdpi.com/article/10.3390/jcm15103898/s1, Figure S1. Visual Analogue Scale (VAS) for pain intensity assessment; Figure S2. Daily prehabilitation programme completion checklist (patient card); Table S1. Participant position and anatomical measurement points for MyotonPRO assessment of mechanical muscle properties. Positions were selected to maximise relaxation of the assessed muscle, and measurement points were localised over the muscle belly based on palpation and brief submaximal activation; Table S2. Test positions and dynamometer placement sites for isometric hip muscle strength assessment using the ActivForce 2 digital dynamometer. The table specifies participant positioning and sensor application sites for hip flexors, abductors, adductors, and extensors; Table S3. Questionnaire items, response options, and analytical coding; Table S4. Instructional materials for the home-based prehabilitation programme; Table S5. Correlations between the compliance index and changes in preoperative clinical and functional outcomes from M1 to M2; Table S6. Associations between compliance index and demographic and clinical variables.

## Abbreviations

The following abbreviations are used in this manuscript:

10MWT	10-m Walk Test
BMI	body mass index
HGS	handgrip strength
HOOS	Hip disability and Osteoarthritis Outcome Score
M1	baseline assessment before prehabilitation
M2	preoperative assessment immediately before total hip arthroplasty, after prehabilitation
M3	postoperative assessment at discharge
STROBE	Strengthening the Reporting of Observational Studies in Epidemiology
THA	total hip arthroplasty
TUG	Timed Up and Go
VAS	Visual Analogue Scale
