# Supplementary material for: Compliance with Home-Based Prehabilitation and Length of Stay After Total Hip Arthroplasty: A Prospective Cohort Study
Source: J Clin Med. 2026 May 19;15(10):3898. doi: 10.3390/jcm15103898 (PMC13206761; doi:10.3390/jcm15103898)
Supplement: Supplementary file 1 [file jcm-15-03898-s001.zip › jcm-4278116-supplementary.pdf]

Compliance with Home-Based Prehabilitation and Length of Stay after Total Hip Arthroplasty: A Prospective Cohort Study

Supplementary Materials

MyotonPRO

Mechanical muscle properties were assessed using the non-invasive digital MyotonPRO device (Myoton AS, Tallinn, Estonia). The device generates brief mechanical impulses with a duration of 15 ms and a force of 0.4 N, applied under a precompression force of 0.18 N. The impulses induce damped oscillations in the tissues located beneath the probe, which are recorded and converted into the following parameters: muscle tone, expressed as oscillation frequency in Hz, stiffness in N/m, and elasticity, expressed as logarithmic decrement. The probe was applied perpendicular to the skin surface, maintaining a deviation within ± 5°, as indicated by the device. After the precompression load was achieved, the device automatically delivered a series of at least five impulses within a single measurement.

Participants remained at rest with maximal muscle relaxation. Measurement points were determined by palpation over the muscle bellies and marked with a skin marker. To confirm the location, the participant was asked to briefly activate the assessed muscle at a submaximal level, after which the measurement was repeated under full relaxation. Measurements were performed on the operated side, and all assessments were conducted by the same assessor. Measurement sites are presented in Table S1 in the Supplementary Materials [1].

Isometric dynamometry of hip muscles

Isometric muscle strength was assessed using the ActivForce 2 digital dynamometer (Activbody, Inc., San Diego, CA, USA). Measurements were performed for four hip muscle groups, including extensors, flexors, adductors, and abductors. For each muscle group, three measurements were conducted under identical conditions and in the same test position, using a consistent verbal instruction and stabilisation of the participant's position to minimise movement compensations. Participant positioning and the dynamometer application site are presented in Table S2 in the Supplementary Materials.

Each test was performed as a maximal voluntary isometric contraction against resistance. Before recording, the participant received instructions regarding the direction of force generation and the procedure for performing the trial. On the command "start", the participant generated maximal force and maintained the isometric contraction until the command "stop", after which the trial ended and a rest period followed. Between consecutive trials, a rest break sufficient for the sensation of fatigue to subside was provided to ensure comparability of subsequent test results, with a minimum duration of 60 seconds. For each trial, device-generated parameters were recorded, including peak force. The highest of the three values was used for analyses, in accordance with the protocol assumptions. All assessments were performed by the same physiotherapist [2,3].

Functional tests

Functional performance was assessed using the 10-m Walk Test (10MWT) and the Timed Up and Go (TUG) test. If the patient used walking aids during the first assessment, the same aids were also used during the second assessment. Walking time was measured with a stopwatch over a distance of 10 m, which allowed calculation of gait speed under steady-paced conditions. The test was performed at the patient's comfortable speed. One measurement trial was conducted.

Before the TUG test, a rest period was provided. The participant started in a seated position on a chair with armrests. On instruction, the participant stood up, walked 3 m at a comfortable speed, turned around, returned to the chair, and sat down again. The duration of the trial was measured with a stopwatch from the initiation of standing up to returning to the seated position. One familiarisation trial was performed before the measurement, followed by one measurement trial.

Hip disability and osteoarthritis outcome score (HOOS)

The Hip disability and osteoarthritis outcome score (HOOS) consists of 40 items divided into five domains: pain (10 items), symptoms, including stiffness and range of motion (5 items), activities of daily living limitations (17 items), sport and recreation function (4 items), and hip-related quality of life (4 items). Responses to all items are provided using a five-point Likert scale (none, mild, moderate, severe, extreme). Each response is scored from 0 (no problems) to 4 (extreme problems). Domain scores were calculated as the sum of item scores within each domain and then transformed to a 0 to 100 scale according to the standard procedure, accounting for the number of completed items. After transformation to the 0 to 100 scale, higher scores indicated better function and fewer symptoms [4].

References

[1] P. E. Muckelt *et al.*, "Protocol and reference values for minimal detectable change of MyotonPRO and ultrasound imaging measurements of muscle and subcutaneous tissue.," *Sci. Rep.*, vol. 12, no. 1, p. 13654, Aug. 2022, doi: 10.1038/s41598-022-17507-2.

[2] R. Vișan, M. Mihajlov, M. A. Luminița, and A. I. G. Baușic, "Muscle Deflection Used as an Assessment Indicator of the Rehabilitation Quality After Total Hip Joint Arthroplasty (THA).," *J. Clin. Med.*, vol. 14, no. 11, May 2025, doi: 10.3390/jcm14113728.

[3] C. Karagiannopoulos, S. Griech, and B. Leggin, "Reliability and Validity of the ActivForce Digital Dynamometer in Assessing Shoulder Muscle Force across Different User Experience Levels.," *Int. J. Sports Phys. Ther.*, vol. 17, no. 4, pp. 669–676, 2022, doi: 10.26603/001c.35577.

[4] W. Glinkowski, A. Żukowska, M. Dymitrowicz, E. Wołyniec, B. Glinkowska, and D. Kozioł-Kaczorek, "Translation, Cross-Cultural Adaptation, and Psychometric Properties of the Polish Version of the Hip Disability and Osteoarthritis Outcome Score (HOOS).," *Medicina (Kaunas).*, vol.

55, no. 10, Sep. 2019, doi: 10.3390/medicina55100614.

Figure S1. Visual Analogue Scale (VAS) for pain intensity assessment. A 10 cm horizontal line anchored by "no pain" (0) and "worst imaginable pain" (10). Participants marked the perceived pain intensity, and the score was calculated as the distance (cm) from the "no pain" anchor to the mark.

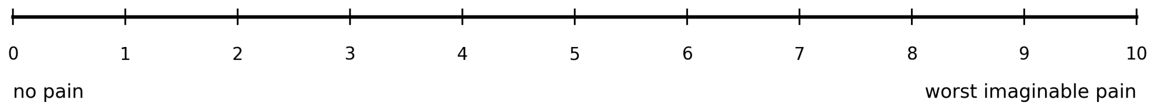

Figure S2. Daily prehabilitation programme completion checklist (patient card). Participants recorded the daily level of exercise completion using five categories (0%, 25%, 50%, 75%, 100%) according to the provided instructions, including shading the corresponding proportion of each circle. Blank fields indicate no exercises performed.

Please shade the entire circle if all exercises were performed on that day.

Please shade 3/4 of the circle if most exercises were performed on that day.

Please shade half of the circle if half of the exercises were performed on that day.

Please shade 1/4 of the circle if less than half of the exercises were performed on that day.

Please leave the field blank if no exercises were performed on that day.

1		16		31		46	
2		17		32		47	
3		18		33		48	
4		19		34		49	
5		20		35		50	
6		21		36		51	
7		22		37		52	
8		23		38		53	
9		24		39		54	
10		25		40		55	
11		26		41		56	
12		27		42		57	
13		28		43		58	
14		29		44		59	
15		30		45		60	

Table S1. Participant position and anatomical measurement points for MyotonPRO assessment of mechanical muscle properties. Positions were selected to maximise relaxation of the assessed muscle, and measurement points were localised over the muscle belly based on palpation and brief submaximal activation.

**Muscle**	**Participant position**	**Measurement points**
Gluteus maximus	Prone, lower limbs in a neutral position, shanks supported with a bolster to reduce tension of the posterior thigh muscles.	Along the line connecting the posterior superior iliac spine and the greater trochanter of the femur, at 50% of the distance between the bony landmarks, over the most prominent part of the muscle belly, with localisation adjusted based on palpation during brief hip extension in the prone position.
Gluteus medius	Side-lying on the contralateral side, tested limb positioned in slight abduction, hip and knee joints in neutral alignment.	Along the line connecting the superior border of the iliac crest (lateral portion) and the greater trochanter, at 50% of the distance, at the point of maximum palpated muscle thickness, confirmed with brief lower limb abduction.
Biceps femoris	Prone, lower limb extended at the knee joint, foot positioned freely beyond the edge of the examination table or supported with a bolster.	On the lateral part of the posterior thigh surface, along the line connecting the ischial tuberosity and the fibular head, at 50% of the distance. Location was confirmed by palpation during brief knee flexion with slight external rotation of the shank, then the measurement was performed under complete muscle relaxation.
Rectus femoris	Supine, with a bolster placed under the knee to reduce tension of the anterior thigh muscles.	On the anterior thigh surface, along the line connecting the anterior superior iliac spine and the superior border of the patella, at 50% of the distance, along the muscle belly axis, confirmed with a brief straight leg raise.
Adductor longus	Supine, lower limb in slight abduction and external rotation, with support under the knee to ensure comfort and relaxation.	On the medial thigh surface, along the line connecting the pubic tubercle and the medial lip of the linea aspera of the femur, at 30% of the distance measured from the pubic tubercle, within the palpated muscle belly. Location was confirmed with brief hip adduction against resistance.

Table S2. Test positions and dynamometer placement sites for isometric hip muscle strength assessment using the ActivForce 2 digital dynamometer. The table specifies participant positioning and sensor application sites for hip flexors, abductors, adductors, and extensors.

**Muscles**	**Position**	**Dynamometer placement site**
Hip flexors	Participant supine, tested limb in a neutral position, with stabilisation of the pelvis and trunk to limit compensatory trunk movement.	Anterior surface of the distal one-third of the thigh, approximately 5 cm proximal to the superior border of the patella, along the limb axis.
Hip abductors	Participant side-lying on the side opposite the tested limb, trunk and pelvis stabilized, tested limb aligned with the abduction movement axis.	Lateral surface of the distal one-third of the thigh, approximately 5 cm proximal to the lateral femoral epicondyle, along the limb axis.
Hip adductors	Participant supine, tested limb flexed at the hip and knee joints, positioned to ensure comfort and relaxation, foot stably supported on the examination table.	Medial surface of the distal one-third of the thigh, approximately 5 cm proximal to the medial femoral epicondyle, along the limb axis.
Hip extensors	Participant prone, lower limbs in a neutral position, with hip and knee extension, without limb rotation.	Posterior surface of the distal one-third of the thigh, approximately 5 cm proximal to the popliteal fossa, avoiding pressure within the popliteal fossa.

Table S3. Author-developed questionnaire items used in the analysis, response options, and coding scheme across study time points. The table includes variable names used in the manuscript, item wording, categorical response options, analytical coding, and the measurement occasion (M1, M2, M3). Abbreviations: M1, baseline assessment before prehabilitation, M2, preoperative assessment immediately before total hip arthroplasty (after prehabilitation), M3, postoperative assessment at discharge.

Variable in the manuscript	Question wording	Response options	Coding for analysis	Time point
Marital status	What is the patient's marital status?	single, married, divorced, widowed	1 = single, 2 = married, 3 = divorced, 4 = widowed	M1
Place of residence	What is the patient's place of residence?	rural area, town/city up to 10000 inhabitants, city 10000 to 50000 inhabitants, city over 50000 inhabitants	1 = rural area, 2 = town/city up to 10000 inhabitants, 3 = city 10000 to 50000 inhabitants, 4 = city over 50000 inhabitants	M1
Education	What is the highest completed level of education?	primary, vocational, secondary, higher	1 = primary, 2 = vocational, 3 = secondary, 4 = higher	M1
Cigarette smoking	Does the patient currently smoke cigarettes?	yes, no	0 = no, 1 = yes	M1
History of joint arthroplasty	Has the patient previously undergone joint arthroplasty (hip or knee)?	no, hip, knee	0 = no, 1 = hip, 2 = knee	M1
Self-rated motivation before surgery	How does the patient rate their motivation to complete the prehabilitation programme before surgery?	scale 1–5	1 = very low, 2 = low, 3 = moderate, 4 = high, 5 = very high	M1
Self-rated preparedness for surgery	How does the patient rate their preparedness for the planned surgery?	scale 1–5	1 = very low, 2 = low, 3 = moderate, 4 = high, 5 = very high	M2

Variable in the manuscript	Question wording	Response options	Coding for analysis	Time point
Self-reported completion of the exercise programme	Does the patient declare completion of the prescribed exercise programme during prehabilitation?	yes, no	0 = no, 1 = yes	M2
Pain during exercises	Was pain present while performing the prescribed exercises?	yes, no	0 = no, 1 = yes	M2
Self-rated current health status compared with pre-treatment status	How does the patient assess their health status on the day of discharge (M3) compared with the status before surgical treatment?	marked improvement, moderate improvement, slight improvement, no improvement, deterioration	1 = deterioration, 2 = no improvement, 3 = slight improvement, 4 = moderate improvement, 5 = marked improvement	M3

Table S4. Author-developed home-based prehabilitation exercise programme. The table lists strengthening and stretching exercises with starting positions, execution instructions, hold times, repetitions, and bilateral performance rules for unilateral tasks.

**Strengthening exercises:**		
1.	Isometric quadriceps setting, strengthens the quadriceps femoris muscle. Starting position: supine. Execution: Press the knee into the surface, hold the contraction for 8 seconds, relax. Perform 10 repetitions with the right and left leg.	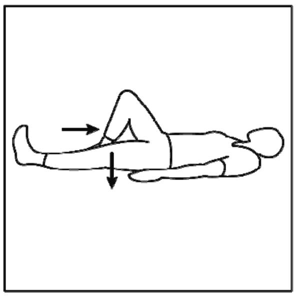
2.	Hip flexion of the lower limb, improves hip flexor strength and increases hip joint range of motion. Starting position: supine. Execution: Flex the lower limb by sliding the heel along the surface up to the pain limit or range-of-motion limitation, return to the starting position. Perform 10 repetitions with the right and left leg.	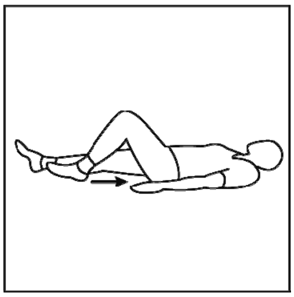
3.	Hip bridge, strengthens the gluteal muscles and hip extensors.<br>Starting position: supine, lower limbs flexed at the hip and knee joints, feet supported on the surface. Execution: Lift the pelvis upward, hold the position for 3 seconds, return to the starting position. Perform 10 repetitions.	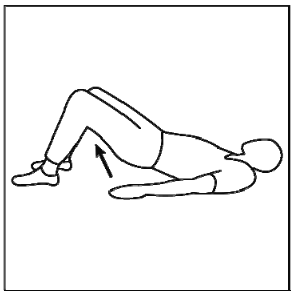
4.	Straight leg raise at the hip joint, strengthens the hip flexors. Starting position: supine. Execution: Raise the straight lower limb, hold for 3 seconds. Slowly lower the lower limb and return to the starting position. Perform 10 repetitions with the right and left leg.	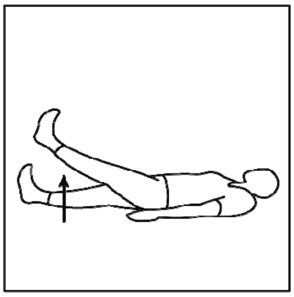
5.	Hip external rotation in side-lying, increases external rotation range of motion, strengthens the external rotators of the hip joint. Starting position: side-lying, lower limbs flexed at the knee joints. Execution: Perform external rotation with the upper leg, hold the position for 3 seconds. Perform 10 repetitions with the right and left leg.	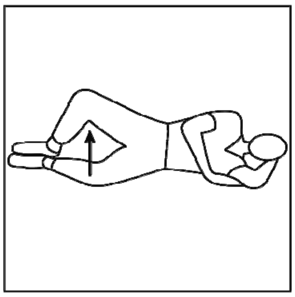

6.	Gluteal strengthening in standing, improves gluteal muscle strength. Starting position: standing with the hands supported on the backrest of a chair in front. Execution: Perform hip extension with the lower limb, hold the position for 3 seconds, return to the starting position. Perform 10 repetitions with the right and left leg.	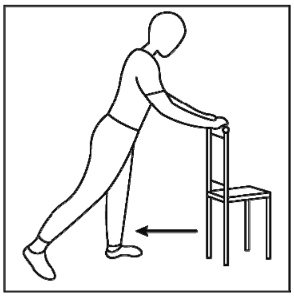
7.	Half squats using a chair, strengthen the quadriceps femoris and gluteal muscles. Starting position: standing with the hands supported on the backrest of a chair in front. Execution: Perform a half squat, return to the starting position. Perform 10 repetitions.	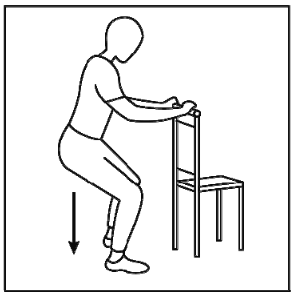
**Stretching exercises:**		
1.	Hip rotations in supine, stretch the internal and external hip rotator muscles.<br>Starting position: supine with the lower limbs flexed at the hip and knee joints, feet supported on the surface. Execution: Lower both lower limbs to the right side, hold the position for 3 seconds, return to the starting position, repeat on the left side. Perform 10 repetitions on each side.	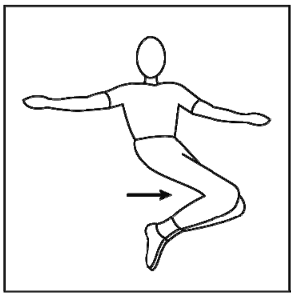
2.	Hip adductor stretch. This exercise aims to improve the flexibility of the hip adductor muscles. Starting position: standing with the feet placed slightly wider than shoulder width. Execution: shift body weight onto the right leg and hold the position for 5 seconds, then repeat on the left leg. Perform 10 repetitions, alternating sides.	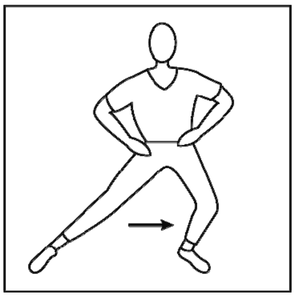
3.	Hip flexor stretching in a standing forward and backward lunge position using a chair, stretches the hip flexor muscles. Starting position: standing with the hands supported on the backrest of a chair, one lower limb in a forward lunge position and the other in a backward lunge position. Execution: Shift body weight forward onto the front limb, hold for 5 seconds, then switch legs and repeat. Perform 10 repetitions.	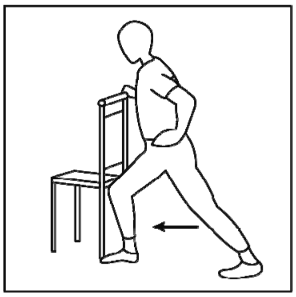

Table S5. Correlations between the compliance index and changes in preoperative clinical and functional outcomes from M1 to M2. Spearman's rank correlation coefficients (rho) and p values are presented for changes in HOOS domains, VAS pain intensity, MyotonPRO parameters, isometric hip muscle strength, and functional test performance (TUG, 10MWT) (n = 40).

**Variable (n=40)**	**ΔM2-M1**			**Compliance with the exercise plan, weighted mean [%]**	
	**Median**	**IQR**	**Min-max**	**Rho**	***p***
HOOS pain [0–100]	40.0	26.25–47.5	10.0–75.0	-0.014	0.9295
HOOS symptoms [0–100]	37.5	25.0–45.0	5.0–75.0	-0.036	0.8269
HOOS activities of daily living [0–100]	33.1	20.59–46.33	4.41–82.35	-0.054	0.7396
HOOS sport and recreation [0–100]	25.0	12.5–28.13	0.0–68.75	-0.261	0.1035
HOOS quality of life [0–100]	25.0	6.25–34.38	0.0–62.5	0.052	0.7490
VAS [0–10]	7.0	5.0–8.0	1.0–10.0	-0.203	0.2082
MyotonPRO, gluteus maximus, frequency [Hz]	11.1	10.45–12.45	9.6–19.0	0.054	0.7397
MyotonPRO, gluteus maximus, stiffness [N/m]	230.0	212.0–259.5	136.0–299.0	0.057	0.7285
MyotonPRO, gluteus maximus, elasticity	2.0	1.57–2.33	1.04–3.05	-0.018	0.9103
MyotonPRO, gluteus medius, frequency [Hz]	15.6	14.15–17.35	12.4–24.0	0.026	0.8722
MyotonPRO, gluteus medius, stiffness [N/m]	277.5	249.0–314.0	219.0–672.0	-0.019	0.9096
MyotonPRO, gluteus medius, elasticity	1.2	1.07–1.31	0.72–3.16	-0.049	0.7621
MyotonPRO, biceps femoris, frequency [Hz]	14.4	12.9–15.8	11.3–28.9	-0.145	0.3735
MyotonPRO, biceps femoris, stiffness [N/m]	272.0	259.0–290.0	178.0–381.0	-0.064	0.6949
MyotonPRO, biceps femoris, elasticity	2.0	1.61–2.57	0.88–3.17	-0.073	0.6553
MyotonPRO, rectus femoris, frequency [Hz]	14.2	12.6–15.25	10.3–18.2	0.063	0.6992
MyotonPRO, rectus femoris, stiffness [N/m]	292.5	249.5–304.5	194.0–388.0	-0.015	0.9260
MyotonPRO, rectus femoris, elasticity	1.9	1.52–2.12	1.36–2.82	-0.262	0.1026
MyotonPRO, adductor longus, frequency [Hz]	12.9	11.65–14.0	9.8–21.1	-0.030	0.8520
MyotonPRO, adductor longus, stiffness [N/m]	265.5	240.0–282.5	202.0–337.0	0.102	0.5291
MyotonPRO, adductor longus, elasticity	1.8	1.5–2.05	1.07–3.77	-0.066	0.6874
Dynamometer, hip extensors, muscle strength [kg]	10.6	7.95–14.45	1.5–30.5	-0.053	0.7441
Dynamometer, hip flexors, muscle strength [kg]	12.5	7.85–17.55	1.9–41.1	0.009	0.9584
Dynamometer, hip adductors, muscle strength [kg]	8.5	5.0–11.9	1.3–19.9	-0.125	0.4412
Dynamometer, hip abductors, muscle strength [kg]	13.7	9.6–20.35	1.7–36.8	-0.050	0.7594
TUG [s]	14.8	10.57–17.38	6.3–58.33	-0.014	0.9311
10MWT [s]	6.8	4.84–8.63	3.86–28.75	0.169	0.2971

Table S6. Association between programme compliance (compliance index) and selected demographic and clinical variables in the study group (n = 40). Data are presented as n (%), median [IQR] (min to max), or Spearman's rho, as appropriate. For categorical predictors, p values refer to between-group comparisons of the compliance index. For continuous predictors, p values refer to Spearman correlation with the compliance index. Statistically significant results are marked with an asterisk ($p < 0.05$).

**Variable**	**Category**	**Study group, n (%) or median [IQR] (min to max)**	**Compliance index, median [IQR] or Spearman rho**	**p**
Sex	female	15 (37.5%)	46.3 [4.9–89.2]	0.0578
	male	25 (62.5%)	9.7 [0.0–56.3]	
Age		63.5 [57.34–71.66] (36–86)	-0.071	0.6647
BMI [kg/m²] (M1)		29.36 [27.34–30.85] (20.76–36.11)	-0.041	0.7999
Marital status	single	3 (7.5%)	23.3 [5.8–24.2]	0.8517
	married	29 (72.5%)	40.3 [0.0–70.0]	
	divorced	3 (7.5%)	44 [17.3–64.8]	
	widowed	5 (12.5%)	13.2 [0.0–93.8]	
Place of residence	rural area	18 (45.0%)	33.8 [0.0–79.5]	0.6426
	town up to 10000 inhabitants	4 (10.0%)	11.7 [0.0–34.8]	
	town 10000–50000 inhabitants	5 (12.5%)	71.6 [0.0–91.1]	
	city over 50000 inhabitants	13 (32.5%)	24.5 [1.6–56.3]	
Education	primary	3 (7.5%)	45.8 [11.5–61.6]	0.7808
	vocational	14 (35.0%)	8.2 [0.0–64.3]	
	secondary	9 (22.5%)	13.2 [1.6–53.2]	
	higher	14 (35.0%)	48.0 [0.0–82.6]	
Operated hip	right	17 (42.5%)	45.8 [12.0–90.0]	0.0177*
	left	23 (57.5%)	5.7 [0.0–53.5]	
History of joint arthroplasty	no	29 (72.5%)	45.8 [0.0–73.7]	0.4800
	hip	11 (27.5%)	23.3 [2.1–43.3]	
	knee	0 (0.0%)	N/A	
Smoking	no	32 (80.0%)	23.4 [0.0–62.5]	0.4107
	yes	8 (20.0%)	55.1 [4.9–81.3]	
Self-rated motivation [1–5]		4 [4–5] (2–5)	0.111	0.4955
Self-rated preparedness [1–5]		4.5 [4–5] (3–5)	0.233	0.1479
Pain during exercises	no	15 (37.5%)	71.7 [27.6–81.0]	0.0127*
	yes	25 (62.5%)	8.3 [0.0–46.0]	

* Statistically significant ($p < 0.05$).
